# Adoption, Domestication, and Alienation: A Case Study of Teacher AI Integration Practices and Their Driving Factors in K-12 Classrooms

**DOI:** 10.3390/bs16050658

**Published:** 2026-04-27

**Authors:** Shixiao Wang, Wenye Li, Shusheng Shen, Weihao Wang, Jian Xiao, Aibin Tang

**Affiliations:** 1 School of Education Science, Nanjing Normal University, Nanjing 210097, China; 220601025@njnu.edu.cn (S.W.); shen@njnu.edu.cn (S.S.); 2 Institute of Education, Nanjing University, Nanjing 210093, China; 3 Department of Educational Administration, East China Normal University, Shanghai 200062, China; 52274110003@stu.ecnu.edu.cn (W.W.); 52284110005@stu.ecnu.edu.cn (J.X.); 4 School of Education, Zhejiang International Studies University, Hangzhou 310023, China; tangaibin@zisu.edu.cn

**Keywords:** generative artificial intelligence, teacher technology practice, practice typology, case study

## Abstract

As generative artificial intelligence (GenAI) tools undergo rapid iteration, the complexity and heterogeneity of teachers’ technology practices in authentic instructional contexts warrant closer empirical scrutiny. Focusing on a public middle school designated as an AI demonstration site in eastern China, this study drew on 17 months of fieldwork that combined critical incident interviews, participant observation, and artifact collection. Systematic thematic analysis yielded four distinct practice types: Implicit Empowerment, Ritualized Enhancement, Transformative Exploration, and Prudent Distancing. The differentiation among these types was traced to the interplay of four dimensions: professional agency, technological cognition, organizational governance, and field culture. Specifically, the professional agency dimension encompasses trade-offs in labor intensity, preservation of professional authority, and continuity of pedagogical habitus; the technological cognition dimension manifests as misalignment of technological empowerment, concerns over output hallucinations, and the narrowing of dialogic value; the organizational governance dimension includes evaluation system orientation, excessive resource consolidation, and a lack of tolerance for innovation failure; and the field culture dimension involves peer practice modeling, team cultural atmosphere, and stakeholder demands. Together, these factors help explain the diversity of teachers’ technology adoption behaviors and offer an empirically grounded framework for understanding the micro-level processes of AI integration into classroom teaching.

## 1. Introduction

Across educational systems worldwide, teachers’ contact with and use of GenAI-based tools (hereafter “AI tools”) has expanded at a striking pace. Yet the latest TALIS (Teaching and Learning International Survey) data show that, in the vast majority of participating countries and regions, AI tool use still clusters around lower-order cognitive tasks—generating practice exercises, organizing materials, or supporting lesson preparation—with little evidence of deeper integration that cultivates higher-order thinking or restructures classroom interaction (OECD, 2025). This pattern persists even in schools with ample resources and well-developed training programs.

Prior work has largely attributed variation in teachers’ technology practices to individual competencies or integration stages, operating on the assumption that positive perceptions of usefulness, ease of use, or integration depth will naturally steer adoption toward more advanced applications (X. Chen et al., 2024). Such a perspective presupposes a linear pathway from “adoption intention” to “application behavior,” treating complex classroom practices as straightforward externalizations of intent. In reality, the path from intention to behavior is anything but direct; a “black box” intervenes between what teachers want to do and what they actually do (Shi et al., 2024). Linear models alone cannot account for why teachers with comparable competencies and equivalent resources end up on divergent practice trajectories. Compounding this limitation, the field’s reliance on cross-sectional surveys and variable-centered models, though useful for isolating determinants of intention, leaves uncharted the dynamic decision making through which teachers move from “wanting to use” to “how to use”—or “why not to use”—in day-to-day classrooms. Understanding the heterogeneity of teachers’ AI practices therefore requires opening this black box to trace how intentions are reconstructed, inhibited, or redirected within particular institutional settings. Grasping these mechanisms calls for a practice-proximate research stance (Brown, 2017)—one that observes, in situ, how teachers negotiate the meaning and limits of AI use within the daily rhythms of instruction, institutional pressures, and collegial interaction. Only such an approach can surface the situated reasoning that standardized instruments tend to miss and illuminate why a single technology takes on strikingly different forms from one teacher to the next.

To this end, the present study adopts a dual-layer analytical approach. The first layer uses the PICRAT model (Kimmons et al., 2020) as a descriptive coordinate system to map the spatial distribution of teachers’ AI practices without imposing a hierarchical ranking. The second layer traces the situated factors behind the observed distribution through systematic thematic analysis of prolonged fieldwork data. This mapping–tracing design is intended to capture practice diversity while also surfacing the contextual mechanisms that shape it. Against this backdrop, two research questions guide the present study:

RQ1: What typical practice types of AI integration do frontline teachers exhibit in their teaching?
RQ2: What factors shape the differentiation of teachers’ AI integration practice types or the superficiality of their integration behaviors?

## 2. Literature Review

### 2.1. The Latent Heterogeneity of Teachers’ Technology Practices

When teachers encounter emerging technologies such as big data analytics and AI tools, their instructional practices tend to diverge in both form and depth. Scholarship has addressed this divergence through three broad theoretical lenses: stage theory, hierarchy theory, and knowledge structure theory. Each foregrounds a different analytic entry point—adoption chronology, integration depth, and knowledge competency—and together they supply the conceptual scaffolding for the present review.

Stage theory, represented by the Diffusion of Innovations Theory (DOI), conceptualizes technology adoption as a dynamic process encompassing knowledge, persuasion, decision, implementation, and confirmation, and accordingly classifies adopters into five categories ranging from “innovators” to “laggards” (Rogers et al., 2014). The perceived attributes central to DOI—relative advantage, complexity, and the like—have demonstrated solid explanatory power for adoption intentions and remain central to accounts of early-adopter behavior (Abdalla et al., 2024; Singh & Strzelecki, 2026). Hierarchy theory focuses on evaluating the depth of technology integration, with the SAMR model and the ICAP framework being two mainstream theories. The SAMR model categorizes teachers’ technology practices into four hierarchical levels from low to high: Substitution, Augmentation, Modification, and Redefinition (Puentedura, 2015). Recent evidence suggests that, despite broadly positive attitudes, teachers’ actual practices tend to cluster at the Substitution and Augmentation tiers, and self-assessments frequently overestimate integration depth (W. Luo et al., 2025). As a complement, the ICAP framework classifies technology use from a cognitive engagement perspective into four modes—Passive, Active, Constructive, and Interactive—further evaluating the differentiation in technology integration levels (Chi & Wylie, 2014; Antonietti et al., 2023). Cross-national survey data confirm that both the frequency and quality of technology integration differ across cognitive levels, and that the capacity of different digital tools to support each ICAP mode is uneven (Maričić et al., 2025), reflecting the latent heterogeneity of technology use. Knowledge structure theory, centered on the TPACK framework, attributes the heterogeneity of teachers’ technology practices to the degree of integration among Technological Knowledge (TK), Pedagogical Knowledge (PK), and Content Knowledge (CK) within specific instructional contexts (Mishra & Koehler, 2006), thus explaining heterogeneity from a knowledge structure perspective (Zou et al., 2022). Empirical work corroborates that teachers’ growth across these knowledge dimensions is strongly shaped by individual and disciplinary characteristics, with the depth of composite knowledge serving as a reliable predictor of higher-order teaching practice (Baran et al., 2019).

Taken together, stage, hierarchy, and knowledge structure theories have laid critical groundwork for mapping differences in teachers’ technology use. Their shared limitation, though, is a reliance on linear scales—“can versus cannot,” “high versus low,” “fast versus slow”—that risk collapsing practice differences into rungs on a competency ladder (Hamilton et al., 2016). The rapid spread of GenAI in schools, however, defies the template of earlier technology cycles. Its generativity, interactivity, and blurred application boundaries place new demands on teachers’ professional self-conceptions, their situated disciplinary cultures, and the institutional norms of their organizations. Because adoption has surged within barely two years and the technology continues to iterate at speed, treating GenAI as just another variable in a competency framework risks underestimating its heightened context dependency. There is, therefore, a pressing need to examine authentic classroom settings in order to identify practice types that capture the contextual complexity of current AI tool use.

### 2.2. Driving Factors of Teachers’ Technology Practices

The Technology Acceptance Model (TAM) and its derivatives (e.g., UTAUT) have anchored most attempts to explain what drives teachers’ technology practices.

In its original form, TAM foregrounds two variables—Perceived Usefulness (PU) and Perceived Ease of Use (PEOU)—centered on an individual’s appraisal of a technology’s instrumental attributes. TAM2 and TAM3 extended the model by incorporating social influence and subjective norms, thereby acknowledging the role of external expectations and peer evaluations (Al-Qaysi et al., 2020). Venkatesh et al. (2003) distilled these expansions into the Unified Theory of Acceptance and Use of Technology (UTAUT), which integrates Performance Expectancy, Effort Expectancy, Social Influence, and Facilitating Conditions into a single framework spanning individual cognition, social pressure, and organizational support. Applied to teachers’ AI tool use, empirical work has largely confirmed the continued explanatory relevance of acceptance-based models. Boughanzai et al. (2026) confirmed through an extended TAM framework that perceived ease of use and usefulness remain the core pathways predicting teachers’ adoption of AI tools, and found that variables such as trust and social influence play critical moderating roles in the formation of adoption intentions. Intrinsic motivation (e.g., perceived enjoyment) has been identified as the strongest predictor of adoption behavior, and teachers from different types of institutions exhibit significant path differences in social norms and performance expectancy (Y. Luo et al., 2026). Furthermore, J. Chen et al. (2025) found, by integrating Task-Technology Fit (TTF) with TAM, that the fit between AI tools and instructional tasks, along with teachers’ innovative attitudes, is a key determinant of their practice intentions, while perceived ease of use serves as a necessary precondition underpinning the entire decision-making process. Taken together, these studies indicate that, once enriched with variables for affect, task characteristics, and organizational context, classical models retain their capacity to identify the principal drivers of teachers’ AI adoption.

While the foregoing models have identified an array of significant predictors and thereby expanded the variable space for explaining adoption motivation, they rest on a “rational actor” assumption—that use intention translates smoothly into actual behavior—and implicitly treat teachers as decision-makers operating in a social vacuum. Some studies do acknowledge that technology decisions are nested within the organizational architecture of schools—shaped by performance accountability, organizational culture, and disciplinary traditions (Song et al., 2025)—but collapsing these contextual forces into single variables in a structural model risks flattening the complex role they play in teachers’ situated deliberation. Recent empirical work beyond the education sector reinforces this concern. Studies of human–AI collaboration in organizational settings have shown that AI adoption influences employee behavior not through direct pathways, but through mediating psychological mechanisms such as identity formation (Xu et al., 2025) and sequential resource depletion (Jung & Kim, 2026), suggesting that the intention–behavior relationship in AI contexts is inherently more complex than classical acceptance models assume. These limitations motivate the present study’s turn toward a frontline case study, using prolonged field observation and iterative inquiry to situate teachers’ micro-level deliberations within the broader dynamics of digital transformation.

### 2.3. An Integrated Analytical Stance: From Descriptive Mapping to Contextual Tracing

The two strands of the literature reviewed above converge on a shared structural gap. On the practice-classification side, frameworks such as SAMR and ICAP offer hierarchical scales that rank technology use from “lower” to “higher” levels. While useful for benchmarking, these scales embed a linear evaluative assumption: practice differences are ultimately reducible to differences in competency, cognition, or adoption stage. Hamilton et al. (2016) identified three specific weaknesses in the SAMR model along these lines—its neglect of instructional context, its foregrounding of technology as product rather than process, and its reliance on a fixed hierarchy that equates higher-level integration with better teaching. Blundell et al. (2022), in a scoping review of 230 SAMR-based studies, reinforced this critique with a striking empirical finding: apparently similar teaching actions were assigned to different SAMR levels across studies, indicating that hierarchical classification becomes unstable when stripped of its instructional context. On the adoption-driver side, TAM and UTAUT have been effective at identifying variables that predict use intention, yet they rest on a smooth transmission assumption—that positive intention leads reliably to actual practice. Teachers, in these models, appear as rational actors deliberating in a social vacuum.

Together, these two limitations point to a core question that existing theories struggle to address: why do teachers with comparable competencies and equivalent access to technological resources end up on divergent practice trajectories? Belland (2009) offered a productive reframing by drawing on Bourdieu’s concept of habitus. He argued that technology integration is not a direct externalization of beliefs, but is instead embedded in durable dispositions shaped by teachers’ accumulated professional experiences. Positive beliefs about technology, in other words, do not automatically translate into deep integration—a finding that subsequent empirical work has confirmed (Ertmer et al., 2012). Bicalho et al. (2023), studying teachers’ ICT use during the pandemic, reached a complementary conclusion: rather than progressing linearly from substitution to redefinition, teachers moved fluidly across SAMR levels in response to shifting contextual demands. These findings collectively suggest that understanding heterogeneity in teachers’ AI practices requires moving beyond competency ladders and intention-behavior chains toward a stance that is closer to the practice itself—one that attends to how teachers negotiate, adapt, and sometimes resist technology within their specific institutional settings.

Guided by this practice-proximate stance, the present study adopts a dual-layer analytical approach that links descriptive mapping to contextual tracing.

The first layer—descriptive mapping—locates practices within a non-hierarchical coordinate space. Rather than ranking teachers on an integration ladder, this layer aims to chart the spatial distribution of their AI practices. For this purpose, we adopt the PICRAT model proposed by Kimmons et al. (2020). PICRAT consists of two intersecting dimensions: the PIC axis captures students’ relationship to the technology (Passive, Interactive, or Creative), while the RAT axis captures the technology’s impact on the teacher’s existing practice (Replacement, Amplification, or Transformation). The resulting 3 × 3 matrix provides an open descriptive space rather than a prescribed progression. This feature is critical for the present study. SAMR, by design, implies that movement from Substitution toward Redefinition represents improvement (Hamilton et al., 2016). PICRAT, by contrast, was developed as “a tool of reflection, not prescription” (Kimmons et al., 2020), allowing researchers to record where practices fall without pre-assigning value to any particular cell. This descriptive openness makes it possible to detect patterns that linear hierarchies would obscure—such as experienced teachers clustering in low-level cells not because of skill deficits, but as a deliberate strategic choice. In addition, PICRAT addresses a limitation of the ICAP framework (Chi & Wylie, 2014), which captures cognitive engagement on the student side but does not systematically account for the teacher’s pedagogical decisions. By incorporating both a student-experience axis and a teacher-practice axis within a single matrix, PICRAT enables the analyst to observe the interplay of the two.

The second layer—contextual tracing—explains why practices cluster where they do. The PICRAT matrix can show that teachers’ practices are distributed unevenly across the coordinate space, but it cannot explain the forces behind that distribution. Why does one teacher remain in the Passive–Replace cell while a colleague with similar qualifications moves toward Creative–Transform? Answering this question requires looking beyond any single integration model and into the institutional ecology of day-to-day teaching—the professional identity negotiations, technological sense-making processes, organizational governance pressures, and collegial dynamics that jointly shape practice choices. This second layer of analysis does not rely on a pre-specified theoretical framework for deductive coding. Instead, it follows the principles of systematic thematic analysis (Braun & Clarke, 2006) to inductively identify the factors that account for practice differentiation, drawing on interview, observation, and artifact data collected through prolonged fieldwork.

Taken together, these two layers constitute the study’s analytical architecture. The first layer uses the PICRAT matrix to code observed teaching episodes and generate a spatial map of practice types, thereby addressing RQ1 (What typical practice types of AI integration do frontline teachers exhibit?). The second layer traces the situated logic behind the observed distribution, thereby addressing RQ2 (What factors drive the differentiation of teachers’ AI integration practice types?). This mapping–tracing design allows the study to describe practice diversity without collapsing it into a competency ranking, while also surfacing the contextual mechanisms that hierarchical models tend to flatten into isolated variables.

## 3. Methodology

Teachers’ AI-related practices are neither the mechanical output of a fixed competency set nor the direct translation of a stated intention; they emerge through ongoing adjustment amid daily instructional routines, institutional demands, and collegial relations. Case study methodology, with its commitment to thick description, is well suited to rendering the organizational ecology that survey data alone struggle to capture (Creswell & Creswell, 2017). It enables the reconstruction of how teachers assign meaning to technology within their specific organizational milieu and documents the strategic choices that emerge through sustained interaction with their environment.

### 3.1. Research Context

This study was conducted at a public junior middle school (School H) in eastern China. As a provincial-level AI education demonstration school, School H is well equipped with digital infrastructure: all regular classrooms are equipped with smart terminals, and a dedicated AI laboratory is available. The school administration provides stable support through administrative measures and project-based management. Regarding faculty capacity, the school supports teachers’ participation in relevant research projects and professional training programs, and most teachers have accumulated practical experience with AI tools through these project-driven initiatives. This environment provides a foundation for observing teachers’ diverse practice behaviors in a technology-rich context. In addition, the researcher and research team were deeply involved in the school’s daily instructional management activities and had established a constructive rapport with relevant administrators, which provided multiple data access channels for obtaining different stakeholders’ understandings of the same phenomena, thereby facilitating triangulation.

### 3.2. Participants

To ensure that the selected cases could precisely address the aforementioned research questions and identify participants capable of providing in-depth responses, this study first conducted Phase 1 sampling based on six months of field observation at the case school. During this period, through the cumulative observation of over fifty lessons, attendance at instructional meetings, and daily interactions, a preliminary understanding of the school’s teachers’ basic patterns of AI tool use was established. Subsequently, purposive sampling was employed to recruit potential interviewees. These included teachers who actively used AI tools in demonstration lessons, those who proactively shared their AI use experiences during informal conversations, and those who explicitly opposed AI tools, ensuring that participants reflected the diversity of the teacher population. To further identify teachers with unique experiences, Phase 2 involved snowball sampling: initial pre-selected teachers were invited to recommend colleagues, thereby discovering potential participants who were not publicly active but possessed specific use experiences in their actual teaching, thus increasing sample heterogeneity. In Phase 3, the formal recruitment stage, the researcher utilized after-school seminars to explain the research purpose, procedures, and anonymity protections to all potential participants, and, adhering to the principles of informed consent and voluntary participation, completed the final confirmation.

Ultimately, 13 formal participants were confirmed, spanning different disciplines, years of teaching experience, and technology proficiency levels. Among them, 3 were administrative personnel responsible for instructional management and informatization, included to provide institutional and managerial perspectives on technology application. In the analysis, data from these three participants were used exclusively as contextual evidence for understanding organizational governance and field culture dynamics (Section 4.2.3 and Section 4.2.4); the four practice types reported in Section 4.1 were derived solely from classroom-level data provided by the ten frontline teachers. The detailed demographic characteristics and background information of the participants are recorded in Table 1.

### 3.3. Data Collection

Preliminary analysis of existing, non-interventional instructional data and anonymized information commenced in March 2024, which is legally exempt from ethical review under national regulations (Article 32, Measures for Ethical Review of Life Sciences and Medical Research Involving Humans, China). Subsequently, formal qualitative data collection involving direct human subjects (e.g., in-depth interviews and participant observations) was conducted strictly following the approval of the Institutional Review Board in December 2024, employing the following three primary methods.

Semi-structured interviews. Using the critical incident technique, one-on-one interviews of 60 to 90 min were conducted with each participant, asking teachers to describe specific instructional episodes in detail to elicit comprehensive narratives and concrete examples of their AI practices (see Table 2 for the interview protocol). All interviews were transcribed verbatim for subsequent analysis.

Participant observation. Observations encompassed 16 formal classroom sessions, as well as informal settings including teaching and research meetings, office discussions, and group chats on social media. The focus was on documenting how teachers and students used technology features, the specific purposes of use, and the interaction processes among teachers, students, and technological entities, with particular attention to the interaction dynamics between teachers and their colleagues.

Artifact collection. Documents systematically collected included the school’s AI implementation plans, teachers’ lesson plans before and after the introduction of AI tools, social media chat logs, and student assignments.

### 3.4. Coding Framework and Coding Process

**Figure 1:**
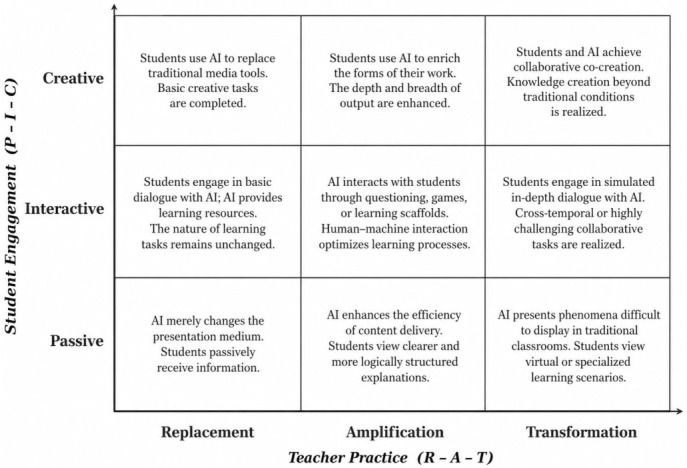
PICRAT-based coding framework for teachers’ AI practices.

The data analysis followed the systematic thematic analysis procedure proposed by Braun and Clarke (2006), assisted by NVivo 12 software for coding. As discussed in Section 2.3, the PICRAT model (Kimmons et al., 2020) served as the coding framework for the descriptive mapping layer of the analysis, providing a non-hierarchical coordinate space defined by student engagement modes (P–I–C: Passive, Interactive, Creative) and technology impact on teacher practice (R–A–T: Replace, Amplify, Transform) (see Figure 1). The contextual tracing layer then drew on inductive thematic coding of interview, observation, and artifact data to identify the factors behind practice differentiation.

To ensure the rigor of the analytical results, the research team organized two members to independently code 20% of the data based on the initial coding scheme, yielding a Cohen’s Kappa coefficient of 0.82, indicating a high level of inter-rater agreement. All coding discrepancies were resolved through collective discussion and evidence comparison, ensuring the reliability of the analytical process. Throughout the analysis, cross-verification was conducted among what was said in interviews, what was observed, and what was documented, thereby achieving triangulation. After preliminary findings were formulated, the researcher revisited four participating teachers to confirm that the typological classification was consistent with their authentic practice experiences.

## 4. Findings

Analysis of the data pointed to discernible differentiation in how teachers engaged with AI tools. At the surface level, the differences showed up in how often and how deeply teachers used AI; at a deeper level, they reflected how teachers navigated competing demands from their professional routines and organizational expectations. Four typical practice types were identified, along with the factors that account for their differentiation.

### 4.1. The Practice Terrain: Four Differentiated Types of Teachers’ AI Applications

**Figure 2:**
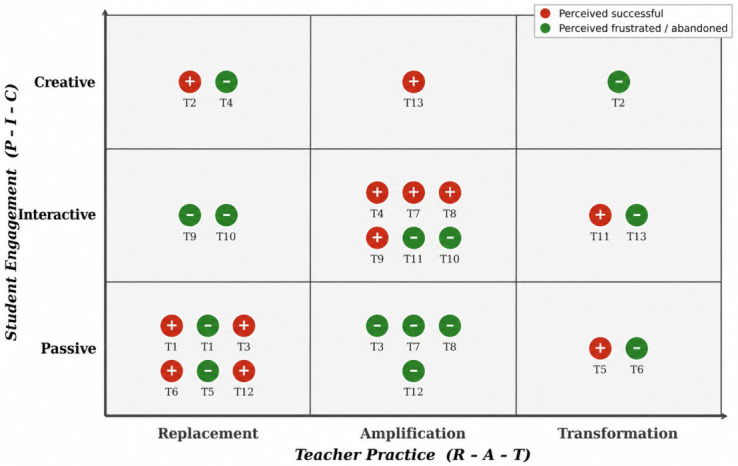
PICRAT-based coding results of teachers’ AI practices.

In-depth analysis of 26 complete teaching episodes reported by the 13 participating teachers revealed a discernible spatial distribution of practices. Figure 2 presents the distribution of these cases within the PICRAT coordinate system, in which red markers represent cases perceived by teachers as successful applications and green markers represent experiences of frustration or abandonment. Triangulating interview data with the case distribution, we distilled the observed practices into four representative types. Each type captures a distinctive way of engaging AI tools and, at the same time, a particular strategic orientation within the constraints of everyday teaching.

#### 4.1.1. Implicit Empowerment

The first type is Implicit Empowerment, represented by teachers T1, T3, and T12. This group predominantly comprises veteran teachers with extensive teaching experience and well-established professional recognition. In their use of AI tools, they demonstrated a distinctive backstaging strategy, strictly confining AI tools to supportive roles in the preparation phase—for example, using AI to generate preliminary instructional materials, which were then screened, revised, and integrated by the teacher, ultimately presented to students in a teacher-led manner. In the classroom foreground, they consistently maintained firm control over the logical thread of instruction, the interpretive authority over knowledge, and the pacing of the lesson, showing no inclination to allow technology to intervene in the restructuring of their instructional design. Interview accounts confirmed that this pattern reflected not resistance to AI, but confidence in their own pedagogical craft. These teachers treated AI as a “digital patch” that boosted the efficiency of an already effective framework—just another tool in a long line of supplementary aids, or as T3 put it, “just a supplementary tool.”

#### 4.1.2. Ritualized Enhancement

The second type is Ritualized Enhancement, represented by teachers T7, T8, T9, and T10, whose technology practices exhibited pronounced contextualized and performative characteristics. In routine teaching, this group tended to exercise restraint in technology use, favoring lower-order support tasks such as knowledge Q&A. However, in scenarios carrying institutional incentives—such as teaching research demonstrations or public evaluation sessions—their technology use shifted to an active display mode, incorporating elements like digital avatar introductions, real-time classroom word cloud generation, and human–AI collaborative task completion, demonstrating a conspicuous “intelligentized” quality. For example, during a district-level open lesson, T8 designed an AI-powered word cloud activity as the lesson opener: students typed keywords into a shared platform, and the AI aggregated responses in real time on the projector screen. The activity generated visible audience engagement and was praised by attending evaluators. However, in a follow-up interview, T8 acknowledged that the word cloud served primarily as a “warm-up device” intended to create a lively classroom atmosphere rather than to scaffold any specific learning objective. The technology practice logic of these teachers represented a highly contextualized strategic choice: in routine instruction, they prioritized efficiency and controllability, concentrating on passive, lecture-centered delivery; in public settings, they proactively responded to the organization’s expectations for “technology demonstration.” What is instructive here is that the behavioral logic amounted to a situational performative strategy: the “innovation” resided in the digital packaging of instruction rather than in any substantive shift in students’ cognitive engagement or depth of processing.

#### 4.1.3. Transformative Exploration

The third type is Transformative Exploration, represented by teachers T2, T4, T11, and T13, who demonstrated a strong internally driven orientation. These teachers’ use of AI tools was more rooted in their own understanding of “what constitutes good teaching,” proactively exploring the possibilities of using AI tools to guide student thinking. For example, teacher T11 configured the AI as a debater holding a specific stance; students were required to identify logical fallacies, evidential gaps, or value presuppositions in the AI’s arguments in real time and generate counter-arguments and supplementary evidence on the spot, thereby prompting students to achieve real-time knowledge generation and sustained depth of thinking through in-depth dialogue with the AI. Using the conventional TPACK framework as an example, these teachers exhibited a clear three-domain overlap in knowledge generation and were willing to apply it in instructional settings for “trial and error” and “inquiry,” viewing technology application as an active expression of agency and a creative transformation of pedagogical philosophy.

#### 4.1.4. Prudent Distancing

The fourth type is Prudent Distancing, represented by teachers T5 and T6, whose technology use exhibited a distinctly defensive logic. On one hand, under the demands of the external environment, they adopted a minimal adoption strategy, bearing surface-level resemblance to the Implicit Empowerment type; on the other hand, they also attempted higher-order applications, yet unlike the Transformative Exploration type, these teachers generally gave low evaluations of the outcomes of such attempts. Interviews indicated that these teachers held highly idealized expectations of AI efficacy, gravitating toward the view that a “truly useful” tool should deliver high-quality instructional outcomes without requiring significant additional learning or effort. When actual outcomes fell short of these expectations, the teachers’ causal attributions gravitated toward the technology’s limitations and immaturity. T5’s experience illustrates this dynamic. After attending a training workshop, T5 attempted to use an AI tool to generate differentiated reading comprehension questions for students at varying proficiency levels. When the generated questions contained factual inaccuracies and failed to align with the curricular sequence, T5 concluded that “the technology is simply not mature enough for real classroom use” and reverted to manually prepared materials, with no subsequent attempt at recalibration. Under the influence of this cognitive structure, teachers felt safe in lower-order applications (Passive–Replace) but became quickly frustrated in higher-order attempts, thereby reinforcing the impression that “technology is more style than substance.” By maintaining distance from technology to ensure professional security, they formed a closed loop of technology value cognition.

### 4.2. Shaping Logic: Multi-Dimensional Forces Behind Teachers’ AI Practice Differentiation

**Figure 3:**
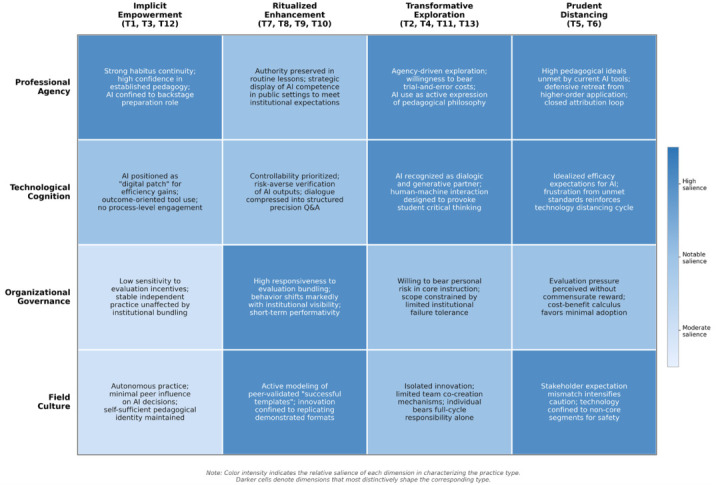
Cross-tabulation of practice types and influencing dimensions.

Building on the typology presented in Section 4.1, the analysis proceeded to identify the factors underlying teachers’ differentiated practice choices. The data revealed that differentiation emerged not from any single cause but from the interplay of four dimensions: professional agency, technological cognition, organizational governance, and field culture. Figure 3 maps these four dimensions onto the four practice types, with color intensity indicating relative salience. Table 3 further details each dimension’s sub-categories and operational definitions. Together, Figure 3 and Table 3 bridge the descriptive mapping of practice positions (RQ1) and the contextual tracing of practice differentiation (RQ2).

Several patterns are worth highlighting. For the Implicit Empowerment type, professional agency was the most defining force: strong habitus continuity and pedagogical confidence kept AI in a backstage role, while organizational and field-cultural pressures exerted comparatively moderate influence. The Ritualized Enhancement type presented a contrasting configuration, with organizational governance—particularly the bundling of technology display with professional development incentives—emerging as the primary shaping force, reinforced by peer modeling of validated “successful templates.” For the Transformative Exploration type, professional agency and technological cognition were jointly salient: these teachers’ willingness to bear trial-and-error costs combined with their recognition of AI as a dialogic partner enabled higher-order practice, though limited institutional failure tolerance constrained its scope. The Prudent Distancing type exhibited high salience across three dimensions simultaneously, suggesting a particularly dense web of reinforcing factors in which high pedagogical ideals, perceived technological immaturity, and stakeholder expectation mismatches converged to sustain defensive retreat.

Across all four types, no single dimension was sufficient to explain any practice pattern. The sections that follow unpack each dimension in detail.

#### 4.2.1. Professional Agency Dimension: An Unformed Collaborative Identity Understanding

Teachers’ technology practice choices were clearly constrained by the internal logic of their professional agency, primarily manifested across three levels—labor costs, professional roles, and teaching experience—which collectively drove teachers to adopt cautious and conservative strategies in AI application.

First, the trade-off of labor intensity constituted the practical boundary of technology adoption. Facing heavy instructional and administrative pressures, teachers’ primary consideration for AI tools was the input–output ratio of time and effort. Interviews revealed that, when making decisions, teachers tended to prioritize the complexity of AI tool calibration over the potential for pedagogical innovation. The high cognitive investment and potential trial-and-error risks inclined teachers to avoid AI tool use that required deep calibration. At the case school, teachers generally preferred to adopt AI application models from social media that were already relatively mature and required no large-scale modifications—models that did not require them to engage in “trial and error” and could “produce results quickly,” thereby maximizing the stability of their workload.

Second, the preservation of professional authority was a key factor constraining the depth of technology intervention. Most interviewed teachers still viewed themselves as the sole source of knowledge interpretation in the classroom, and the intervention of AI tools was readily perceived as a potential undermining of their professional discourse authority. Although some views held that AI tools cannot replace teachers’ unique position in value guidance, this study observed that teachers exhibited a distinct defensive psychology regarding the diminishment of authority at the level of knowledge explanation. Consequently, teachers tended to confine AI tools to auxiliary roles such as reading aloud and question generation, reinforcing rather than replacing their own authority. This strategy was particularly pronounced in routine instruction. Because routine lessons emphasize lecture-based teaching and progress control, teachers often worried that the intervention of technology would disrupt their established instructional rhythm, and even believed that technology had no place in such scenarios. Compared with the use of technology for atmosphere creation in public lessons, the preservation of professional authority in routine lessons narrowed the role of AI tools to that of preparatory instruments.

Finally, the continuity of pedagogical habitus solidified teachers’ path dependency in practice. Teachers with stable pedagogical efficacy, especially veteran teachers with mature teaching styles, often faced the dilemma of misalignment between their established knowledge systems and new tools. Interview data indicated that mature instructional systems were difficult to restructure in the short term to accommodate AI tools. For example, teacher T12 was accustomed to establishing a sense of classroom control through frequent movement and interaction within the physical classroom space; this embodied participatory behavior was highly dependent on the teacher’s guidance of instructional rhythm. AI tools, however, tend to support individual student interaction, and when students’ attention shifted from teacher guidance to screen-based interaction, the instructional connection originally established through physical spatial movement was altered. This shift effectively compressed the teacher’s space for bodily expression, dissolving the experience of classroom control.

#### 4.2.2. Technological Cognition Dimension: An Unformed Human–Machine Trust

In the process of human–machine collaboration, because teachers’ technological understanding remained in a stage of dynamic adjustment, the human–machine trust relationship exhibited notable fragility, directly affecting the breadth of application and the logic of interaction within the instructional field. This played out across three areas: the understanding of technological empowerment, concerns about output content, and the narrowing of dialogic value.

First, the understanding of technological empowerment was limited to a tool-positioning focused on outcome-based output. Interviews revealed that most teachers generally harbored an “out-of-the-box” expectation of technology; the empowerment of AI tools was often equated with the immediate usability of their functional outputs, rather than their role as mediating guides within the instructional process. For instance, during lesson preparation, teachers primarily used AI tools to rapidly generate structured lesson plans and seldom utilized them for simulation or rehearsal of specific instructional processes. This outcome-oriented tool perspective confined AI to the automation of auxiliary labor, simplifying it into a resource provider that substitutes for teachers’ repetitive tasks.

Second, concerns about output hallucinations added redundant application burdens. In scenarios involving public instruction or evaluative contexts, teachers tended to exhibit a pronounced risk-averse tendency. In interviews, one teacher candidly stated: “I’d rather spend extra time verifying than bear the uncertainty in the classroom” (T7). Observations also showed that some teachers subjected AI-generated content to secondary verification using traditional search tools, or preset fixed response pathways and compressed dialogue turns in intelligent agent design to mitigate potential risks. This authentically reflected teachers’ core concern for technological controllability, while imperceptibly increasing their cognitive load.

Finally, the simplified logic of AI interaction narrowed the educational potential of dialogue. Although policies encourage deep human–machine collaboration, in classroom practice, such dialogue was often reduced to structured “precision Q&A.” In the practices of the case school, AI tools were predominantly used to answer specific, clear-cut knowledge-based questions, exhibiting a characteristic of “answering equals dialogue.” While this application highlighted the immediacy of feedback, it overlooked the divergent and generative qualities that dialogue itself should possess. A further reflection is that such application may weaken the pedagogical value inherent in classroom silence and pauses. Compared with a teacher’s pedagogically wise silence and hesitant reflective guidance, the instant feedback driven by AI tools, while pursuing personalized forms, may imperceptibly reinforce the standardization of knowledge production.

#### 4.2.3. Organizational Governance Dimension: An Unformed Empowering Responsibility-Sharing Mechanism

In the process of advancing AI empowerment, an effective mechanism for risk-sharing and responsibility distribution between organizational management and teacher practice has yet to be established. Three areas stood out: evaluation indicator guidance, the resource consolidation mindset, and the lack of innovation tolerance.

First, evaluation criteria steered the AI empowerment effort toward visible manifestations. The current incentive mechanisms at the case school were highly focused on high-visibility technology behaviors; for example, in public lessons, demonstration classes, and teaching research activities, the use of AI tools was frequently regarded as a core indicator of instructional innovation. However, there was a lack of attention to low-visibility, high-adaptability applications in routine classrooms. Instructional innovation and AI practice were often institutionally bundled, linking technology practice to professional development opportunities such as title promotion and merit awards. Even teachers who had not yet developed an intrinsic understanding of technology’s value chose to adopt specific technology labels under institutional pressure to meet compliance requirements. Observations showed that this orientation predisposed teachers’ technology practice behaviors toward short-termism and performativity, thereby securing benefits within the evaluation system.

Second, resource consolidation increased the cognitive costs of technology adaptation. The case school had made sustained investments in digital infrastructure, integrating multiple instructional support systems and providing teachers with basic training and technical consultation. Because AI technology iterates rapidly and differs from existing systems in both technical architecture and instructional logic, teachers still needed to undertake individualized calibration based on disciplinary characteristics and classroom needs in their specific applications. Observations indicated that this adaptation process required teachers to invest additional time in content screening and workflow optimization, objectively increasing the cognitive load of lesson preparation. As a result, most teachers opted for symbolic use in highly controllable, low-interaction instructional segments to avoid the risks and costs associated with system complexity. The result was that, despite the apparently rich resources at the school level, the absence of a unified support system aligned with the specific characteristics of AI tools actually inhibited teachers’ willingness to explore the higher-order transformation quadrant (e.g., Creative–Transform).

Finally, the dearth of tolerance for failure suppressed the shared responsibility for technological transformation. The case school actively cultivated an institutional environment encouraging exploration, providing policy space and positive incentives for teachers’ AI-enabled instructional innovation through designated research projects, resource support, and platforms for showcasing outcomes. While the current support system was relatively systematic in distilling and disseminating successful experiences, it had not yet established a normalized mechanism for experience debriefing and process-oriented support regarding “failures” and other unanticipated outcomes encountered during the exploratory process. In this context, teachers in high-stakes instructional scenarios tended to imitate “successful templates” that had already been validated and to avoid applying AI in core instructional segments. As a result, AI use was largely not a deep transformation of AI based on one’s own pedagogical logic, but was strategically confined to non-critical, low-risk instructional activities to ensure that established instructional objectives were not jeopardized.

#### 4.2.4. Field Culture Dimension: An Unformed Shared Vision for Innovation

Teachers’ technology practice behaviors were not the acts of isolated agents; yet, a shared vision oriented toward instructional transformation has not yet been formed among the multiple stakeholders. This was primarily manifested in three aspects: peer practice modeling, team cultural atmosphere, and stakeholder demands.

First, the relational field had not yet formed a shared understanding of AI-empowered teaching. Within the relational field of teachers’ daily interactions, the understanding of AI-empowered teaching was often simplified into a negative contrast with existing pedagogical models. When an individual teacher proposed a new technology application idea, the proposition was readily interpreted as an implicit critique of traditional instructional practices, swiftly transforming technology discussions into value-stance disputes. Because accepting a new approach was perceived as negating prior practice, field members might adopt a defensive posture upon feeling that their professional identity and work habitus were being challenged. Interviews revealed that current teacher AI practices were largely advanced through individual experiences, with innovative attempts exhibiting fragmented characteristics, even implicitly forming mutually isolated “innovation islands.”

Second, the team field had not yet established a normalized co-creation mechanism. Effective instructional innovation often begins with immature conceptual embryos that require team collaboration, iterative refinement, and the infusion of collective wisdom to be transformed into stably implementable plans. However, the current teaching and research collaboration model still has room for optimization in supporting the growth of such “nascent ideas.” Some interviewed teachers indicated that, when they proposed preliminary AI application concepts, the absence of a normalized collaborative research process meant that innovators often had to bear the full-cycle responsibility from plan design to technical implementation on their own. Under the pressure of such high costs, teachers were more inclined to propose low-risk, easy-to-implement incremental improvements, ensuring they could satisfy the formal requirement for “AI elements” in teaching and research activities without bearing the uncertainty and responsibility burden associated with conceptual innovation.

Finally, in the regional field, a shared vision among multiple stakeholders had not yet been formed. Surrounding AI-enabled instructional practice, there existed multiple and not entirely symmetrical interest demands among parents, students, teachers, and external academic discourses. Parents prioritized the stability of academic achievement; students focused on the subjective experience of the interactive process; and external discourses emphasized the “disruptive” role of technology. Facing these differentiated expectations, teachers needed to seek balance within the interstices of multidimensional demands when applying AI tools. Particularly when a single lesson failed to deliver the so-called “disruptive effects,” teachers, to avoid being criticized for “formalism” or “not truly understanding AI,” instead tended to confine technology to passive, controllable, non-core instructional segments, thereby achieving the greatest common denominator among competing stakeholder interests. While this strategy sacrificed the deeper potential of AI tools, it also represented a rational—albeit reluctant—choice to maintain professional security within the interstices of multiple demands.

## 5. Discussion and Conclusions

Through a longitudinal case study of an AI demonstration school, this study identified four typical practice types of teachers’ AI applications: Implicit Empowerment, Ritualized Enhancement, Transformative Exploration, and Prudent Distancing. Further analysis revealed that the formation of these four practice types is shaped by four interacting dimensions: professional agency, technological cognition, organizational governance, and field culture. In the professional agency dimension, teachers generally prioritize labor intensity trade-offs, preservation of professional authority, and continuity of existing pedagogical habitus, inclining technology use toward low-risk, high-controllability pathways. In the technological cognition dimension, most teachers narrow the empowerment value of AI to summative outputs, while heightened vigilance toward algorithmic hallucinations further compresses the application space. In the organizational governance dimension, the school’s evaluation system bundles technology use with professional development opportunities yet lacks tolerance mechanisms for failure. In the field culture dimension, conceptual conflicts among peers lead to “innovation islands,” and the competing expectations of parents, students, and policy discourses place teachers in a predicament regarding higher-order technology application.

Before discussing each of these findings in detail, it is useful to state this study’s core contribution explicitly. As outlined in Section 2.3, the dual-layer analytical approach—descriptive mapping followed by contextual tracing—was designed to avoid the evaluative assumptions embedded in linear integration models. Three propositions emerged from this analysis that qualify prevailing theoretical assumptions. First, similar observable AI practices can reflect fundamentally different underlying motivations. Second, ostensibly “higher-level” technology use may be performative rather than genuinely transformative. Third, apparently “lower-level” use may represent a rational response to institutional constraints rather than a competency deficit. Taken together, the study’s primary theoretical contribution is not a new integration model but a contextual mechanism that explains why teachers with comparable competencies and resources end up on divergent practice trajectories. The four practice types and four explanatory dimensions function as an empirically grounded analytical lens—one that foregrounds the situated interplay of agency, cognition, governance, and culture rather than ranking teachers on a linear adoption scale. The following discussion examines how these propositions relate to prior research.

### 5.1. Discussion of Teachers’ Differentiated Technology Practice Types

A central finding is that teachers’ technology practices with AI tools are not uniform but irreducibly diverse. This observation carries implications for how mainstream theories frame technology adoption.

Qualifying the linear hierarchy assumption. As argued in the literature review, prevailing theories share an implicit premise: practice differences are rooted in individual variation in competency, willingness, or cognitive structure, with “higher-order” use treated as inherently superior. Hamilton et al. (2016) critiqued this premise in the SAMR model, and Blundell et al. (2022) demonstrated its empirical instability. Our data point in the same direction but go further: in a context where AI tools are already widely available, practice differentiation may be better understood as a response to organizational constraints than as a reflection of individual competency levels. Teachers in this study seldom transcended their institutional settings to arrive at the idealized transformative applications that normative models envision. Empirically, both the present study and Song et al. (2025) identified an “ideal” practitioner type oriented toward instructional reconstruction, with considerable behavioral overlap between the two samples. Crucially, however, integration depth did not map neatly onto motivation. The Ritualized Enhancement teachers (e.g., T7, T8, T9, T10) also engaged in higher-order technology practice behaviors—such as incorporating real-time word clouds and human–AI collaborative tasks—yet their underlying motivation was oriented toward institutional display rather than pedagogical transformation (see Section 4.1.2). Classifying teachers solely by integration depth, as SAMR-based approaches tend to do, thus risks mislabeling strategic behavior as developmental lag.

Extending the understanding of performative technology use. The coding results confirm earlier systematic reviews: the bulk of documented practices place students in a passive, resource-receiving role. Where the present findings extend prior work is in documenting a sizeable group of teachers who did engineer student–technology interaction and achieved measurable instructional enhancement—behaviors that, in SAMR or ICAP terms, would readily qualify as “higher-level” integration. Close case observation, however, revealed that much of this interactive practice was animated by an aspiration to cultivate a personal image as an “AI representative” rather than by a commitment to instructional redesign. This pattern resonates with Belland’s (2009) argument that positive technology dispositions do not necessarily translate into substantive integration. Even when teachers do not genuinely endorse the technology, they may—under institutional pressure—repurpose it as a tool for professional display.

Reinterpreting “implicit” and “distancing” practices. The data also surfaced an implicit practice type, confirming Barnes and Tour’s (2026) observation that teachers sometimes conceal AI use. However, our findings diverge from their account regarding the mechanism. Barnes and Tour attributed concealment to concerns about appearing “unprofessional” or “lazy.” In the present case, concealment was instead associated with confidence in one’s own pedagogical command: these veteran teachers (e.g., T1, T3, T12) simply folded AI into an existing repertoire of support tools while preserving a firmly teacher-led format (see Section 4.1.1). Earlier typologies have modeled teachers as fully rational agents whose practices follow deterministically from competency profiles. For example, Choi et al. (2026) argued, based on a “teaching experience–AI proficiency” two-dimensional framework, that experienced teachers can critically adapt AI outputs while novice teachers tend toward passive dependence. The Ritualized Enhancement teachers identified here challenge this prediction: they possessed mature experience and high AI proficiency—conditions that should, in theory, precipitate higher-order use—yet their actual application remained surface-level, largely confined to opening hooks or closing summaries in public lessons.

A further pattern concerns the assumed alignment between pedagogical progressiveness and technological progressiveness. The Prudent Distancing teachers (T5, T6) illustrate that even pedagogically ambitious teachers may resist AI, precisely because their high expectations collide with the technology’s current limitations (see Section 4.1.4). This finding converges with Ertmer et al.’s (2012) observation that beliefs and practices often diverge, but specifies a mechanism: it is not belief weakness that produces resistance, but belief strength meeting technological immaturity.

The overarching lesson is that higher-order behavior need not originate from a transformative motive, nor does lower-order practice automatically signal a competency deficit. The former may be a performative strategic response; the latter may be a rational safeguarding of instructional certainty. As Bicalho et al. (2023) demonstrated in a different context, teachers move across integration levels in response to situational demands rather than progressing along a fixed trajectory. Any account of teachers’ technology practices must therefore attend to the organizational ecology that gives those practices their meaning.

### 5.2. Discussion of the Influencing Factors of Teachers’ Technology Practices

A second overarching finding is that teachers’ AI-related practice choices are shaped not by any single factor but by the interactive dynamics of professional agency, technological cognition, organizational governance, and field culture. Structurally, this aligns with the “internal beliefs–external environment” framework familiar in the literature, yet the present analysis enriches it in several respects.

Professional agency: from idealized beliefs to situated rationality. Earlier studies have typically traced teachers’ boundary-keeping—whether defending professional authority or perpetuating habitual practice—to a relatively stable “teaching belief” or “professional identity.” The picture that emerged from our fieldwork is less idealized. This finding extends Belland’s (2009) habitus-based argument in a specific direction: beliefs functioned less as fixed commitments than as flexible dispositions that shifted with circumstance. For example, T12’s insistence on maintaining physical classroom control through bodily movement (see Section 4.2.1) was not an abstract pedagogical conviction but a habituated practice pattern that AI-mediated interaction threatened to disrupt. A related contribution is the foregrounding of labor intensity trade-offs. Under the dual burden of administrative and instructional demands, teachers’ primary calculus for technology investment was cost rather than philosophy—a pattern consistent with the practical rationality that Bourdieu’s concept of habitus predicts (Belland, 2009) but rarely documented in the specific context of AI integration.

Technological cognition: beyond perceived usefulness. The prevailing explanatory framework—perceived usefulness and ease of use—centers on appraisals of tool efficacy. Sun et al. (2024) confirmed that teachers’ adoption willingness for AI is highly dependent on these two variables. Our findings confirm that perceived usefulness matters, but extend the analysis by revealing a deeper layer: teachers’ understanding of AI depends on how they position themselves in relation to the technology, not merely on whether they find it efficient. This positioning remained unstable at the current stage of AI diffusion. This positioning proved fluid and, at this stage of AI diffusion, broadly fragile. This fragility of human–machine positioning resonates with findings from organizational behavior research. Xu et al. (2025), studying employees’ AI collaboration in workplace settings, identified AI identity—comprising dependence, emotional energy, and relatedness—as a critical mediator of behavioral outcomes. The teachers in the present study, however, had not yet developed a stable AI identity of this kind; their positioning of AI remained tentative and largely instrumental, which helps explain why classroom applications were compressed into lower-order functions. Because teachers tended to overlook the dialogic and generative affordances of AI—treating it as a provider of finished answers rather than as a partner for open-ended inquiry—classroom applications were compressed into one-directional Q&A or resource generation. This pattern is consistent with Blundell et al.’s (2022) finding that SAMR-based categorizations become unstable without attention to teachers’ prior practice baselines: what counts as “amplification” versus “replacement” depends on whether the teacher conceptualizes AI as a dialogue partner or as an answer machine.

Organizational governance: the supportive-condition paradox. The importance of school-level factors for technology adoption is well established. Zhu (2013) identified open school culture as foundational for innovative technology applications; Vanderlinde et al. (2015) emphasized the facilitative role of explicit ICT policies; Håkansson Lindqvist (2019) found that cultivating a “safe trial-and-error” environment stimulated teachers’ exploratory willingness. The present study confirms that these supportive conditions matter. However, it extends prior work by identifying a structural paradox that an emphasis on provision alone tends to overlook. AI tools remain in a phase of rapid iteration and functional immaturity, generating outputs marked by high uncertainty. Teachers in this study inevitably experienced repeated calibration failures and even instructional disruptions during use. Such exploration is cognitively demanding and inherently risky, requiring institutional support for risk sharing. Yet the school’s governance focused on immediately visible outcomes and offered little recognition for the unsuccessful attempts that inevitably accompany exploratory work (see Section 4.2.3). This finding extends Hamilton et al.’s (2016) critique of context-free integration models into the organizational domain: it is not enough to provide resources and encouragement; institutions may also need to absorb the costs of failure if they wish to see teachers move beyond the Passive–Replace quadrant.

Field culture: peers as constrainers, not only enablers. Prior work has noted that colleagues function as both enablers and constrainers of technology practice, with knowledge flows channeled through a handful of key individuals while most teachers remain passive recipients. The present study extends this observation by documenting how, in the specific context of AI adoption, peer interactions were permeated by implicit value-stance tensions. Proposals for new AI applications were readily interpreted as critiques of established teaching methods, converting technology discussions into identity disputes (see Section 4.2.4). Whereas earlier research has painted team culture in a largely facilitative light—through shared norms, modeling, and collective meaning making—this study found team culture operating more as a brake on technology-driven change than as a catalyst for co-creation. Concerning stakeholder demands, the findings converge with Lewin and Luckin (2010) and Li et al. (2025): the benefits of AI-enabled instruction are unevenly distributed across stakeholder groups, and the unresolved tension among competing expectations constitutes an additional constraint on teachers’ practice choices.

### 5.3. Limitations

Several limitations should be acknowledged. First, this study was conducted at a single public middle school designated as a provincial-level AI demonstration site in eastern China. This school benefits from above-average digital infrastructure, sustained administrative support, and regular faculty training. The practice types and influencing factors identified here emerged within this relatively favorable institutional context and may manifest differently in schools with fewer resources or weaker policy support. The findings should therefore be read as empirically grounded propositions rather than as universally generalizable conclusions.

Second, the sample of 13 participants, while appropriate for an in-depth qualitative case study aimed at analytical generalization (Creswell & Creswell, 2017), does not permit statistical generalization to the broader teacher population. Future research could test whether the proposed typology and its associated explanatory dimensions hold across larger samples, diverse school types, and different cultural contexts.

## Figures and Tables

**Table 1:** Demographic characteristics of participating teachers (*n* = 13).

Variable	Category	*n*	Percentage (%)
Gender	Male	8	61.5%
	Female	5	38.5%
Discipline	STEM	6	46.2%
	Humanities	7	53.8%
Teaching Experience	Novice (<5 years)	4	30.8%
	Experienced (6–15 years)	5	38.5%
	Veteran (>15 years)	4	30.8%
Administrative Role	Regular teacher	10	76.9%
	Mid-level administrator/School leader	3	23.1%
AI Tool Proficiency	Beginner	4	30.8%
	Intermediate	7	53.8%
	Advanced	2	15.4%

**Table 2:** Interview protocol (excerpts).

Key Interview Questions
Q1: What AI tools do you currently use most frequently? Please describe a work or teaching scenario in which you are most accustomed to using these tools.
Q2: Please describe in detail one teaching application experience that you consider most effective.
Q3: Please recall a situation in which you attempted to apply AI but felt it was awkward, uncomfortable, or the outcome fell short of expectations.
Q4: Was there an occasion when a task made you repeatedly waver between a traditional approach and an AI-empowered approach? What were the circumstances?
Q5: During lesson preparation or work tasks, are there scenarios in which you regularly use AI but would not mention it in public presentations?
Q6: Please share an experience in which students’ use of AI-generated content produced unexpected feedback in the classroom. What response strategies did you adopt at the time?
Q7: Do you typically showcase your AI-empowered outcomes to others? Among the feedback you received, what made you feel supported, and what made you feel pressured or constrained?

**Table 3:** Coding results of factors influencing teachers’ AI practices.

First-Order Dimension	Second-Order Dimension	Description
Professional Agency	Labor intensity trade-off	Teachers’ comparison between the learning costs invested in AI tools and the resulting instructional benefits.
	Professional authority preservation	Boundary-control behaviors adopted by teachers to safeguard their instructional status and pedagogical certainty in AI applications.
	Pedagogical habitus continuity	The filtering effect of teachers’ established instructional pathways and practice styles on AI tools.
Technological Cognition	Technological empowerment misalignment	Misunderstanding in teachers’ perception of the specific functional positioning of AI tools within the instructional workflow, affecting the scope of AI tool application.
	Output content concerns	Teachers’ concerns about the accuracy of AI-generated information and its potential instructional risks, affecting the depth of AI tool adoption.
	Dialogic value narrowing	Teachers’ narrow understanding of the pedagogical value of AI dialogue, affecting the cognitive level of human–machine collaborative potential.
Organizational Governance	Evaluation indicator guidance	The shaping effect of organizational assessment criteria and incentive mechanisms on teachers’ technology behaviors, affecting the form in which AI tools are presented.
	Resource consolidation mindset	The school’s tendency to consolidate existing digital resources, reflecting the intensity of institutional intervention.
	Lack of innovation tolerance	The organization’s level of acceptance toward unexpected outcomes in exploratory practices, defining the safety boundary for teachers to attempt new pathways.
Field Culture	Peer practice modeling	The implicit influence of peer groups’ technology use frequency and evaluations on individual teachers, reflecting the imitation effect within the field.
	Team cultural atmosphere	The shared attitude toward AI-driven change within teaching and research teams, constituting the psychological background influencing teachers’ exploratory willingness.
	Stakeholder demands	Teachers’ adaptive adjustments to AI application in the process of meeting the instructional quality expectations of parents, students, and other stakeholders.

## Data Availability

The data presented in this study are available upon request from the corresponding author due to privacy and ethical restrictions, as the qualitative data contain information that could compromise the privacy and anonymity of the research participants.

## References

[B1-behavsci-16-00658] Abdalla A. A., Bhat M. A., Tiwari C. K., Khan S. T., Wedajo A. D. (2024). Exploring ChatGPT adoption among business and management students through the lens of diffusion of Innovation Theory. Computers and Education: Artificial Intelligence.

[B2-behavsci-16-00658] Al-Qaysi N., Mohamad-Nordin N., Al-Emran M. (2020). Employing the technology acceptance model in social media: A systematic review. Education and Information Technologies.

[B3-behavsci-16-00658] Antonietti C., Schmitz M.-L., Consoli T., Cattaneo A., Gonon P., Petko D. (2023). Development and validation of the ICAP Technology Scale to measure how teachers integrate technology into learning activities. Computers & Education.

[B4-behavsci-16-00658] Baran E., Canbazoglu Bilici S., Albayrak Sari A., Tondeur J. (2019). Investigating the impact of teacher education strategies on preservice teachers’ TPACK. British Journal of Educational Technology.

[B5-behavsci-16-00658] Barnes M., Tour E. (2026). Teachers’ use of generative AI: A ‘dirty little secret’?. Language and Education.

[B6-behavsci-16-00658] Belland B. R. (2009). Using the theory of habitus to move beyond the study of barriers to technology integration. Computers & Education.

[B7-behavsci-16-00658] Bicalho R. N. D. M., Coll C., Engel A., Lopes de Oliveira M. C. S. (2023). Integration of ICTs in teaching practices: Propositions to the SAMR model. Educational Technology Research and Development.

[B8-behavsci-16-00658] Blundell C. N., Mukherjee M., Nykvist S. (2022). A scoping review of the application of the SAMR model in research. Computers and Education Open.

[B9-behavsci-16-00658] Boughanzai F., Ouhassan Y., Majdoubi R., Hadjoudja A. (2026). A comprehensive structural equation modeling analysis of factors influencing teacher acceptance of AI in education through an extended TAM framework. Education and Information Technologies.

[B10-behavsci-16-00658] Braun V., Clarke V. (2006). Using thematic analysis in psychology. Qualitative Research in Psychology.

[B11-behavsci-16-00658] Brown J. P. (2017). Teachers’ perspectives of changes in their practice during a technology in mathematics education research project. Teaching and Teacher Education.

[B12-behavsci-16-00658] Chen J., He X., Ning Y., Wijaya T. T., Liu J. (2025). Task/Technology Fit or Technology Attraction? The Intentions of STEM Teachers to Use AI Technologies for Teaching Innovation. The Asia-Pacific Education Researcher.

[B13-behavsci-16-00658] Chen X., Hu Z., Wang C. (2024). Empowering education development through AIGC: A systematic literature review. Education and Information Technologies.

[B14-behavsci-16-00658] Chi M. T. H., Wylie R. (2014). The ICAP framework: Linking cognitive engagement to active learning outcomes. Educational Psychologist.

[B15-behavsci-16-00658] Choi S., Jeong S., Park S., Kim Y., Han I. (2026). Analyzing teacher–AI interaction patterns across teacher experience and AI proficiency in student-centered lesson design. Teaching and Teacher Education.

[B16-behavsci-16-00658] Creswell J. W., Creswell J. D. (2017). Research design: Qualitative, quantitative, and mixed methods approaches.

[B17-behavsci-16-00658] Ertmer P. A., Ottenbreit-Leftwich A. T., Sadik O., Sendurur E., Sendurur P. (2012). Teacher beliefs and technology integration practices: A critical relationship. Computers & Education.

[B18-behavsci-16-00658] Hamilton E. R., Rosenberg J. M., Akcaoglu M. (2016). The substitution augmentation modification redefinition (SAMR) model: A critical review and suggestions for its use. TechTrends.

[B19-behavsci-16-00658] Håkansson Lindqvist M. (2019). School leaders’ practices for innovative use of digital technologies in schools. British Journal of Educational Technology.

[B20-behavsci-16-00658] Jung J., Kim M. J. (2026). The impact of artificial intelligence adoption on cybersecurity behavior: A crucial role of corporate ethics. Current Psychology.

[B21-behavsci-16-00658] Kimmons R., Graham C. R., West R. E. (2020). The PICRAT model for technology integration in teacher preparation. Contemporary Issues in Technology and Teacher Education.

[B22-behavsci-16-00658] Lewin C., Luckin R. (2010). Technology to support parental engagement in elementary education: Lessons learned from the UK. Computers & Education.

[B23-behavsci-16-00658] Li M., Vale C., Tan H., Blannin J. (2025). Factors influencing the use of digital technologies in primary mathematics teaching: Voices from Chinese educators. Education and Information Technologies.

[B24-behavsci-16-00658] Luo W., Gao M., Yang Y., He H., Wu Y., Shang Q., Li H. (2025). Early childhood digital pedagogy: Multiple case studies of collective teaching practices in Shanghai kindergartens. Early Childhood Education Journal.

[B25-behavsci-16-00658] Luo Y., Zhou G., Cui Y. (2026). Understanding generative artificial intelligence adoption in higher education faculty: Evidence from Chinese universities and technical and vocational colleges. Education and Information Technologies.

[B26-behavsci-16-00658] Maričić M., Anđić B., Mumcu F., Rokos L., Vondruška J., Weinhandl R., Lavicza Z., Špernjak A. (2025). Evaluating the quality of technology integration across seven European countries with the ICAP Technology Scale. Journal of Computers in Education.

[B27-behavsci-16-00658] Mishra P., Koehler M. J. (2006). Technological pedagogical content knowledge: A framework for teacher knowledge. Teachers College Record.

[B28-behavsci-16-00658] OECD (2025). Results from TALIS 2024: The state of teaching.

[B29-behavsci-16-00658] Puentedura R. R. (2015). SAMR: A brief introduction.

[B30-behavsci-16-00658] Rogers E. M., Singhal A., Quinlan M. M. (2014). Diffusion of innovations. An integrated approach to communication theory and research.

[B31-behavsci-16-00658] Shi L., Ding A.-C., Choi I. (2024). Investigating teachers’ use of an AI-enabled system and their perceptions of AI integration in science classrooms: A case study. Education Sciences.

[B32-behavsci-16-00658] Singh S., Strzelecki A. (2026). Academics as adopters of generative AI: An application of diffusion of innovations theory. Education and Information Technologies.

[B33-behavsci-16-00658] Song Z., Qin J., Jin F., Cheung W. M., Lin C. H. (2025). A case study of teachers’ generative artificial intelligence integration processes and factors influencing them. Teaching and Teacher Education.

[B34-behavsci-16-00658] Sun F., Tian P., Sun D., Fan Y. (2024). Pre-service teachers’ inclination to integrate AI into STEM education: Analysis of influencing factors. British Journal of Educational Technology.

[B35-behavsci-16-00658] Vanderlinde R., Aesaert K., Van Braak J. (2015). Measuring ICT use and contributing conditions in primary schools. British Journal of Educational Technology.

[B36-behavsci-16-00658] Venkatesh V., Morris M. G., Davis G. B., Davis F. D. (2003). User acceptance of information technology: Toward a unified view. MIS Quarterly.

[B37-behavsci-16-00658] Xu J. Q., Wu T. J., Duan W. Y., Cui X. X. (2025). How the human–Artificial Intelligence (AI) collaboration affects cyberloafing: An AI identity perspective. Behavioral Sciences.

[B38-behavsci-16-00658] Zhu C. (2013). The effect of cultural and school factors on the implementation of CSCL. British Journal of Educational Technology.

[B39-behavsci-16-00658] Zou D., Huang X., Kohnke L., Chen X., Cheng G., Xie H. (2022). A bibliometric analysis of the trends and research topics of empirical research on TPACK. Education and Information Technologies.

